# Immunoglobulin G4‐related disease in a dog

**DOI:** 10.1111/jvim.15624

**Published:** 2019-10-26

**Authors:** Lydia J. Colopy, Kai‐Biu Shiu, Laura A. Snyder, Anne C. Avery, Emily D. Rout, A R. Moore

**Affiliations:** ^1^ Veterinary Emergency Service Veterinary Specialty Center Middleton Wisconsin; ^2^ Veterinary Services Marshfield Labs Marshfield Wisconsin; ^3^ Department of Microbiology, Immunology, and Pathology, College of Veterinary Medicine and Biomedical Science Colorado State University Fort Collins Colorado

**Keywords:** eosinophilia, IgE, IgG‐D, immunofixation

## Abstract

Immunoglobulin G4‐related disease (IgG4‐RD), which affects many organ systems, has been recognized as a distinct clinical entity in human medicine for just over a decade but has not been previously identified in dogs. In humans, IgG4‐RD is characterized by diffuse IgG4‐positive lymphoplasmacytic infiltrates that commonly lead to increased serum concentrations of IgG4 and IgE, peripheral eosinophilia, tumorous swellings that often include the parotid salivary glands, obliterative phlebitis, and extensive fibrosis. Herein we describe the diagnosis, clinical progression, and successful treatment of IgG4‐RD in an 8‐year‐old female spayed Husky mixed breed dog. Immunoglobulin G4‐related disease should be considered as a differential diagnosis for dogs with vague clinical signs, lymphoplasmacytic swellings, restricted polyclonal gammopathy, eosinophilia or some combination of these findings.

**Abbreviations**
AGE: agarose gel electrophoresis
CZE: capillary zone electrophoresis
IF: immunofixation
IMGT: Immunogenetics Information System
MCH: major histocompatibility
MUM1: multiple myeloma 1/interferon regulatory factor 4 protein
SPE: Serum Protein Electrophoresis
PARR: PCR for antigen receptor rearrangement
RI: reference interval

## 1 CASE PRESENTATION

An 8 year old female spayed Husky mixed breed dog initially was presented to the primary care veterinarian for evaluation of lethargy, mild hyporexia, intermittent soft feces, vomiting, and coughing. Physical examination at that time disclosed a mildly thin body condition and low‐grade periodontal disease, but no other clinically relevant abnormalities. Thoracic auscultation was normal, the abdomen was soft and nonpainful, and no orthopedic or neurological abnormalities were noted.

Serum biochemical analysis (Beckman Coulter AU 680) performed at Marshfield Laboratories (Waukesha, WI) identified hyperproteinemia of 10.4 g/dL (reference interval [RI], 5.0‐8.3 g/dL) characterized by hyperglobulinemia of 8.0 g/dL (RI, 2.0‐3.8 g/dL). Serum albumin concentration was slightly decreased at 2.4 g/dL(RI, 2.6‐4.0 g/dL). Additionally, a mild increase in serum AST activity (171 U/L; RI, 18‐86 U/L) and mild decrease in serum GGT activity (1 U/L; RI, 3‐19 U/L) were present. Serum electrolyte concentrations were normal except for mild hyponatremia (140 mmol/L; RI, 141‐159 mmol/L). Complete blood count (CBC, Sysmex XT‐2000iV) was normal except for moderate eosinophilia of 6.08 × 10^3^ cells/μL (RI, 0.0‐1.3 × 10^3^ cells/μL) and mild basophilia of 0.68 × 10^3^ cells/μL (RI, 0.0‐0.1 × 10^3^ cells/μL). The total leukocyte count was 16.9 ×10^3^ cells/μL, (RI, 4.0‐18.2 × 10^3^ cells/μL). Moderate hematuria (2+; RI, negative) and proteinuria (300 mg/dL; RI, negative) were identified on a voided urine sample (Mission Urine Reagent Strips, ACON Laboratories).

**Figure 1:**
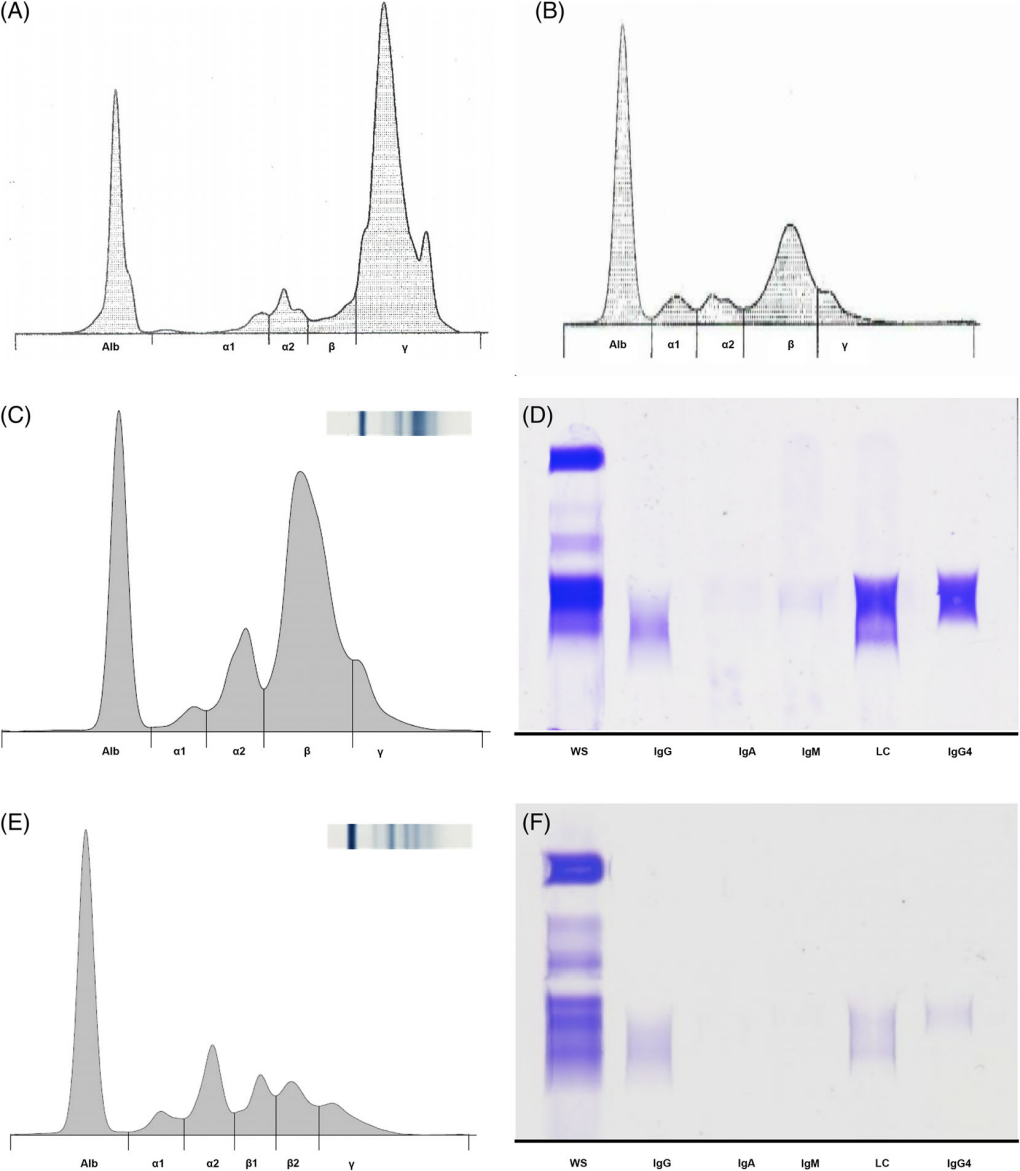
Electrophoresis and immunofixation of serum and urine from a dog. A, Initial serum profiles demonstrated a restricted band in the beta‐globulin region in serum capillary zone electrophoresis. B, Urine protein electrophoresis was also performed and demonstrated a broad protein band within the beta‐globulin fraction. Agarose gel‐based serum protein electrophoresis, C, and immunofixation electrophoresis, D, were performed and identified the wide beta‐globulin band as composed of IgG4. After treatment, the marked polyclonal gammopathy had resolved and the IgG4 band had reverted to a more normal morphology in both serum protein electrophoresis, E, and immunofixation (F)

Serum capillary zone protein electrophoresis (CZE, Sebia Capillarys 2 Flex Piercing, see Supporting Information) identified a large spike in the beta_2_‐gamma globulin region, a second smaller amplitude peak in the cathodal end of the gamma globulin region and a broad beta‐gamma peak that induced beta‐gamma bridging (Figure 1A). The restricted pattern of migration and the magnitude of the spike raised suspicion for a monoclonal gammopathy in a polyclonal base. A urine protein electrophoresis (agarose gel electrophoresis, Sebia Hydrasys System, see Supporting Information) also was performed. A broad band in the beta‐globulin region that mirrored the large spike in the serum was observed and raised concern for monoclonal light chains (Bence‐Jones proteinuria) as well as mild albuminuria (Figure 1B).

**Figure 2:**
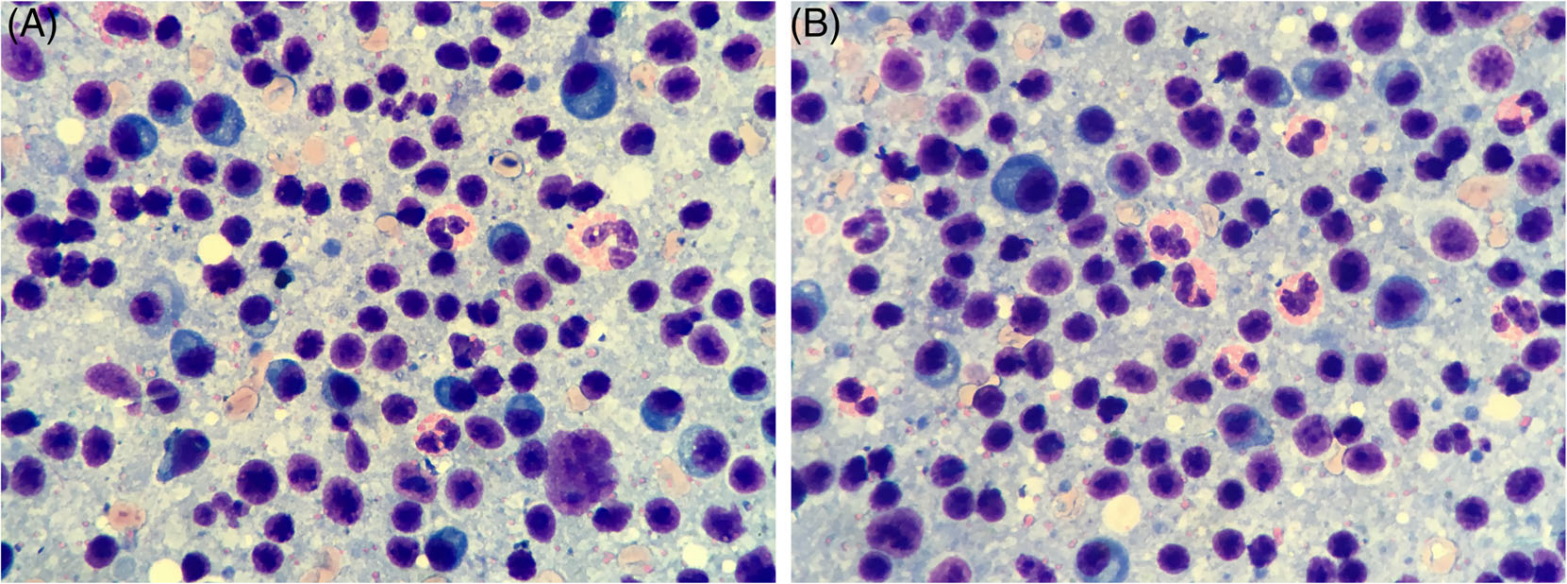
Fine‐needle aspirates of lymph node from a dog. The sample contained a mixed lymphoid population with a notable plasmacytosis as well as low numbers of eosinophils, nondegenerate neutrophils, and rare mast cells. Wright‐Giemsa. Original magnification 100×

The patient was re‐evaluated by the primary care veterinarian after 2 weeks of progressive inappetence and persistent coughing and vomiting. The physical examination again was largely unremarkable. Thoracic radiographs were normal. An abdominal ultrasound examination identified mild bilateral medial iliac lymphadenopathy. Both lymph nodes were mildly enlarged (right, 21 × 9 mm, left, 24 × 8 mm), rounded, and hypoechoic. Fine‐needle aspirates were obtained from both lymph nodes as well as from the ultrasonographically normal‐appearing spleen. Cytology of the spleen indicated lymphoid reactivity and eosinophil‐predominant extramedullary hematopoiesis. Cytology of the lymph nodes identified marked plasmacytosis as well as low numbers of eosinophils, nondegenerate neutrophils, and rare mast cells (Figure 2).

The patient was referred for further evaluation and treatment of possible multiple myeloma. Physical examination again was unremarkable, and no peripheral lymphadenopathy was identified. The dog was normotensive (Doppler blood pressure 110 mmHg) and no lytic lesions were identified on survey spinal radiographs (including the vertebrae, dorsal spinal processes, and olecranon processes). A CBC disclosed persistence of eosinophilia (7.58 × 10^3^ cells/μL; RI, 0.0‐1.3 × 10^3^ cells/μL) and basophilia (0.79 × 10^3^ cells/μL; RI, 0.0‐0.1 × 10^3^ cells/μL). A right humeral bone marrow aspirate was performed to assess for possible plasmacytosis. Bone marrow cytology findings included a myeloid‐to‐erythroid ratio of approximately 2:1 with approximately two‐thirds of the myeloid lineage being eosinophils or eosinophilic hematopoietic precursors. In addition, well‐differentiated plasma cells, found both individually and within small aggregates, accounted for approximately 4% of the total nucleated cell population.

Because of the unexplained eosinophilic bone marrow infiltration and peripheral eosinophilia, additional diagnostic testing was performed to eliminate infectious or inflammatory causes of disease. A Baermann fecal flotation was negative for lungworm ova. A PCR panel was negative for Ehrlichia, Anaplasma, and Neorickettsia.

To further characterize the gammopathy, serum protein electrophoresis and immunofixation (IF) were performed at Colorado State University (serum protein agarose gel electrophoresis [AGE], Sebia Hydrasys System, Sebia France, see Supporting Information).^1^, ^2^ A similar electrophoretic pattern to that observed by serum CZE was detected (Figure 1C). Routine Immunotyping that identifies IgG heavy chain, IgA heavy chain and IgM heavy chain and light chains was performed. In addition, an extended panel to identify canine IgG4 (see Supporting Information, Figure 1D, Donaghy D, Moore A.R. Identification of Canine IgG4 by Immunofixation and Commercially Available Antisera. In Review, *Vet Immunol Immunopathol*) also was performed. The patterns of the AGE and IF were consistent with polyclonal gammopathy and marked IgG4 hypergammaglobulinemia.

Two weeks later, the patient was noted to have developed bilateral chemosis and conjunctival hyperemia, firm swollen parotid salivary glands, and enlarged mandibular lymph nodes. A repeat CBC (Siemens Advia 120, Madison WI) disclosed mild leukocytosis (16 × 10^3^ cells/μL; RI, 4.0‐15.5 × 10^3^ cells/μL) due to persistent eosinophilia (6240 cells/μL; RI, 0‐1200 cells/μL). Serum biochemistry (Beckman Coulter AU680, Madison WI) identified persistent hyperproteinemia (8.6 g/dL; RI, 5.0‐7.4 g/dL) due to hyperglobulinemia (6.0 g/dL; RI, 1.6‐3.6 g/dL). Mild hypoalbuminemia (2.6 g/dL; RI, 2.7‐4.4 g/dL) and moderately increased AST activity (117 IU/L; RI, 15‐66 IU/L) also were present. Urinalysis disclosed persistent proteinuria (100 mg/dL; RI, Negative) with a specific gravity of 1.010 (RI, 1.015‐1.050). A urine protein : creatinine ratio was 1.097 (RI, 0.000‐1.000). Abdominal ultrasound examination at this time also identified moderate splenomegaly with heterogenous echotexture and mildly enlarged left medial iliac lymph node (0.77 cm). Fine‐needle aspirate samples of the spleen were of low cellularity, but cytology indicated eosinophilic hematopoiesis with rare plasma cells. Fine‐needle aspirate and cytology of the mandibular lymph nodes disclosed plasmacytosis with eosinophilic inflammation and extramedullary hematopoiesis.

**Figure 3:**
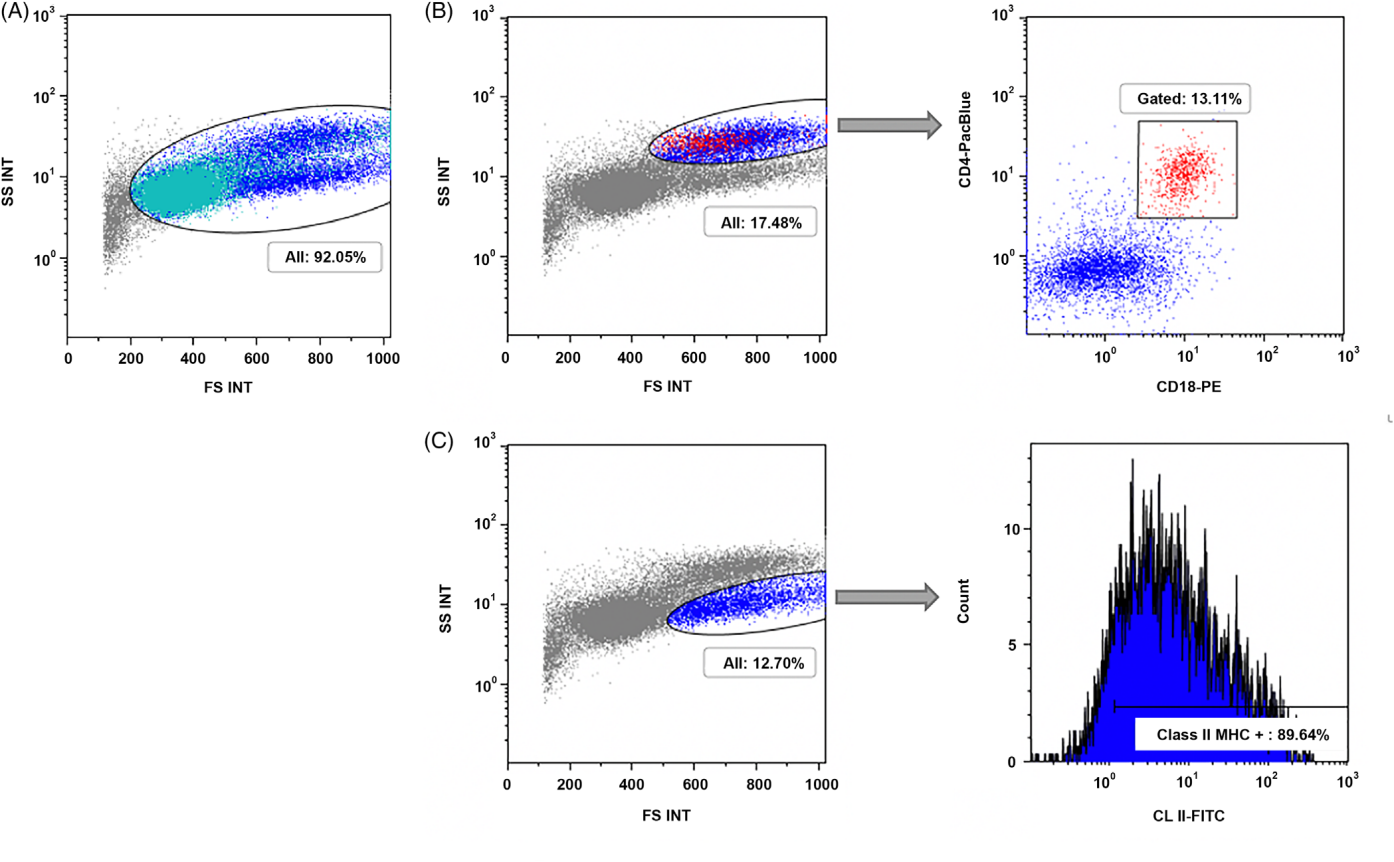
Flow cytometric immunophenotyping of the lymph node aspirate of a dog. A, The size plot with forward scatter (FS) on the horizontal axis and side scatter (SS) on the vertical axis shows a population of small‐sized CD21+ B cells and CD5+ T cells (both colored in green). There are 2 populations (dark blue), which are larger in size with different side scatter properties. All cells in this aspirate express the pan‐leukocyte antigen CD45 (not shown). B, The leukocyte population with high side scatter includes a small percentage of CD4+ CD18+ CD5‐ neutrophils (red). The remainder of cells in this population express CD45 and absent to low levels of CD18. This population may be consistent with eosinophils. C, The leukocyte population with high FS and low side scatter expressed CD45 and approximately 90% of the cells expressed class II MHC. This population may be consistent with plasma cells. MHC, major histocompatibility

Flow cytometry of a splenic sample (Clinical Immunology Laboratory at Colorado State University; see Supporting Information) indicated a normal distribution of lymphocytes, a population of neutrophils, and cells expressing CD‐45 that lacked other markers. These cells were thought to be either eosinophils or mast cells. Flow cytometry of a lymph node aspirate identified a normally distributed population of lymphocytes as well as 2 other populations: a CD45‐expressing population likely to be eosinophils or mast cells, and a class II major histocompatibility (MHC) and CD45‐expressing population likely to be plasma cells (Figure 3). In the authors’ experience (ACA, EDR), confirmed plasma cells (based on multiple myeloma 1/interferon regulatory factor 4 protein [MUM1] expression and morphology) can have similar flow cytometry scatter properties and express CD45 and class II MHC. A PCR for Antigen Receptor Rearrangement (PARR) assay (Clinical Immunology Laboratory at Colorado State University; see Supporting Information) performed on the lymph node aspirate was not consistent with clonality.

The patient was started on 10 mg/kg doxycycline PO q24h, 2.2 mg/kg carprofen PO q24h, neomycin‐polymixin B‐dexamethasone ophthalmic ointment OU q12h, and 0.5 mg/kg enalapril PO q24h. Within 1 week, the polyuria and polydipsia had improved substantially and the patient was transitioned from carprofen to prednisone initially at 1.5 mg/kg PO q24h, tapered to 1.25 mg/kg PO q24h after 1 week and finally to 1 mg/kg PO q24h after another week. At re‐evaluation 1 month later, the ocular changes as well as enlargement of the parotid salivary glands and mandibular lymph nodes had resolved completely. In addition, the patient's serum protein concentrations had returned to normal (TP, 6.8 g/dL; RI, 5.0‐7.4 g/dL; globulin, 3.4 g/dL; RI, 1.6‐3.6 g/dL). The prednisone dosage again was decreased to 0.5 mg/kg PO q24h and after 1 month at this dosage, the patient's clinical signs had not recurred and serum protein concentrations remained normal (TP, 5.9 g/dL; RI, 5.0‐7.4 g/dL; globulin, 2.6 g/dL; RI, 1.6‐3.6 g/dL). Moderate splenomegaly however persisted but the parenchymal echotexture had returned to normal. The prednisone was discontinued and after 1 month, the patient remained clinically normal and serum protein concentrations remained within the reference range (TP, 5.6 g/dL; RI, 5.0‐8.3 g/dL; globulin, 2.5 g/dL; RI, 2.0‐3.8 g/dL). Both AGE and IF results returned to a normal electrophoretic pattern with notably less prominent labeling for IgG4 (Figure 1E,F).

**Figure 4:**
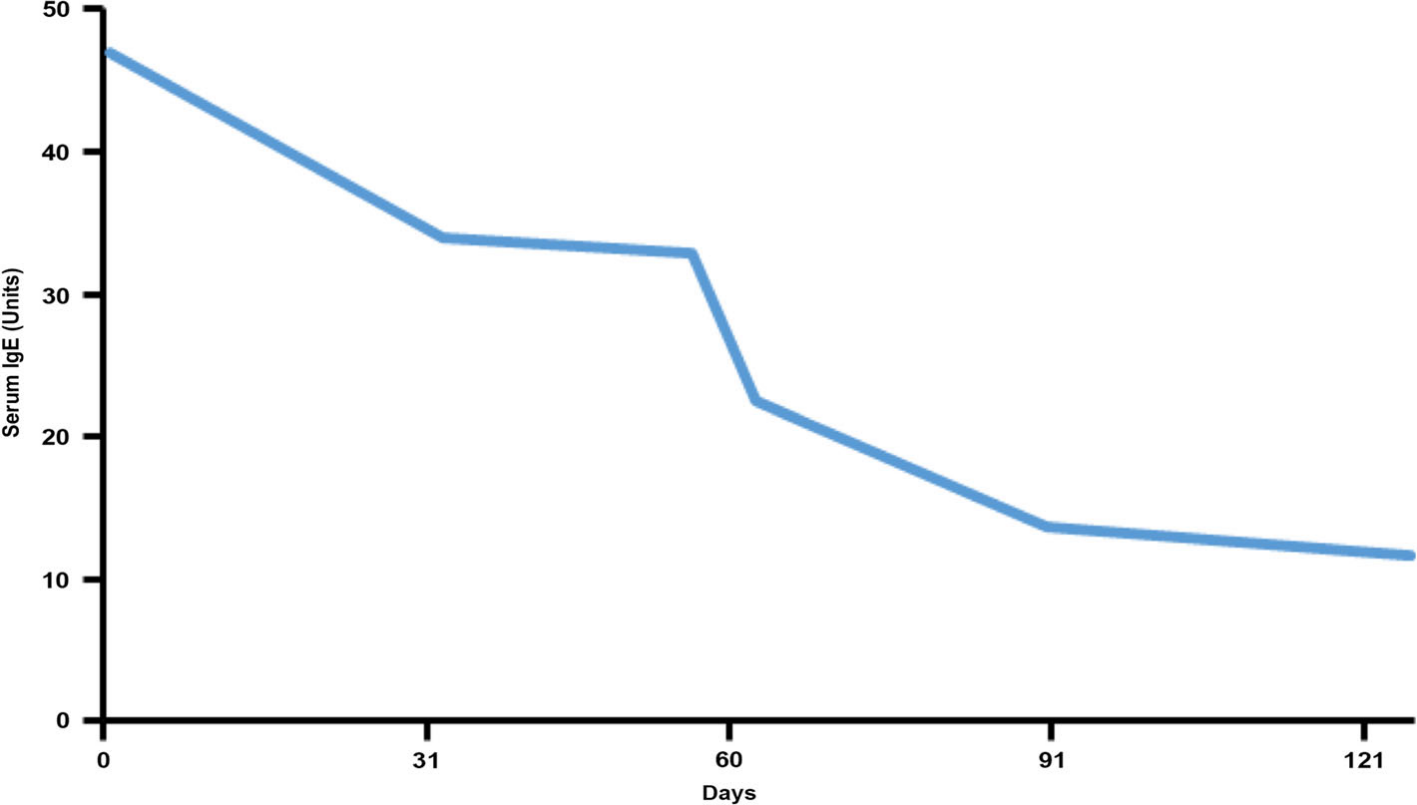
Serum IgE as determined by ELISA from a dog. All samples were assayed within the same run and expressed in units of average serum IgE concentration in 3 normal canine control samples

Serum IgE concentration was measured on archived serum from multiple time points using a commercially available ELISA kit (Immunology Consultants Laboratory, Inc. Portland, OR, see Supporting Information). The IgE results were expressed in units relative to concurrently run control serum. A markedly increased serum IgE concentration at initial diagnosis and more than 4‐fold decrease were observed over the course of evaluations, but all results were markedly increased when compared to normal control patients (Figure 4).

## 2 DISCUSSION

We are aware of a single case of suspected immunoglobulin G4‐related disease (IgG4‐RD) in a dog, which ultimately could not be confirmed.^3^ It is unclear if any of the eosinophilic granuloma cases previously reported in Husky dogs share a common pathogenesis with IgG4‐RD, but the cases do describe involvement of mass‐like lesions around the head or abdominal organs, fibroplasia and lymphoplasmacytic inflammation.^4^, ^5^, ^6^ Ours is the first reported case with clinical support for a diagnosis of IgG4‐RD in a dog.

Because of the use of different evolving and mutually overlapping nomenclature systems, the literature and nomenclature on canine IgG subclasses can be confusing.^7^, ^8^, ^9^, ^10^ We use the current International Immunogenetics Information System (IMGT) database nomenclature of IgG4 to refer to the IgG subclass identified by serum protein purification and DNA sequencing (accession number AF354267, initially named IgG‐D).^9^, ^10^ Concurrently, a 2 subclass system is used for production of commercially available polyclonal IgG antibodies.^10^, ^11^ The “anti‐IgG1” and “anti‐IgG2” antibodies from this system show variable cross‐reactivity with fractions from the 4 subclass systems depending on the testing platform and system used and this 2 subclass system should not be considered congruent with IgG1 and IgG2 of the 4 subclass systems.^10^, ^11^ Ultimately, human IgG4 and canine IgG4 display similar response to physiologic stimuli and binding to F_c_ gamma receptors and are considered similar for the purposes of this work.^10^, ^11^, ^12^, ^13^, ^14^, ^15^

Immunoglobulin G4‐related disease was first described as a distinct entity in the human medical literature as a sclerosing (autoimmune) pancreatitis associated with IgG4 lymphoplasmacytic (IgG4‐producing plasma cells) infiltration and fibrosis of the pancreas.^16^ It subsequently has been identified in many organs, including pancreas, salivary gland, periorbital tissue, retroperitoneum, kidney, biliary tract, lungs, thyroid glands, and other organs.^17^ Affected organs have non‐neoplastic tumoral swellings characterized by a diffuse lymphoplasmacytic infiltrate with IgG4‐positive plasma cells, extensive storiform fibrosis, and obliterative phlebitis.^17^ Cases of IgG4‐RD also have expanded populations of follicular T‐helper cells, cytotoxic CD4+ T cells, IgG4‐positive mast cells and clonal expansion of IgG4‐producing plasma cells.^18^, ^19^ Concurrent with organ involvement, diagnostically useful peripheral eosinophilia, increased serum IgE concentration, and increased polyclonal serum concentrations of IgG4 are commonly found.^17^, ^19^ The IgG4 antibodies in affected human patients appear to target annexin A11 in an autoimmune manner, but the exact pathogenesis of the disease is uncertain because IgG4 typically is thought to have anti‐inflammatory functions.^20^ Other investigations indicate that IgG4‐RD may be a paraneoplastic response.^21^

Tumoral swellings in the parotid salivary gland developed weeks after documentation of a monoclonal gammopathy in our case and may represent a reactive process secondary to the underlying IgG4 disorder. The observed mass‐like lesions were not histologically evaluated, but cytologic evaluation of the lymph nodes, spleen and bone marrow indicated a cellular response consistent with the expected IgG4‐RD cellular findings. The number of IgG4‐positive plasma cells and presence of fibrosis and obliterative phlebitis could not be evaluated by cytology in our case. Histologic evaluation ideally would have been performed to eliminate neoplasia, evaluate for fibrosis and phlebitis, and aid in the diagnosis of IgG4‐RD.

Eosinophilia and increases in serum IgE concentration both have been suggested as markers of IgG4‐RD in humans.^19^ Although our case initially was suspected to have multiple myeloma, the peripheral and infiltrative eosinophilia as well as markedly increased serum IgE concentration were inconsistent with this diagnosis. Screening for non‐IgG4‐RD causes of eosinophilia was performed, but did not detect an agent that could readily explain the eosinophilia. The serum IgE concentration decreased in response to treatment, as expected with IgG4‐RD. Additional studies should evaluate whether or not serum IgE concentration can be used as a marker of response to treatment in the dog.

The initial serum protein electrophoresis identified a high‐concentration and restricted protein band in the beta‐gamma globulin region, which mimicked a monoclonal gammopathy. Immunoglobulin G4 typically is found in very low concentrations in health and may not be readily apparent on routine SPE of the dog.^11^ In humans, increased concentrations of polyclonal IgG4 commonly are found as a restricted‐appearing protein band by routine SPE that gives the appearance of a monoclonal protein.^22^ High‐concentration monoclonal gammopathies typically have a suppressive effect on non‐neoplastic immunoglobulin concentrations.^23^, ^24^ This results in a monoclonal peak standing within what would otherwise be considered hypogammaglobulinemia. This finding is in contrast with the broad polyclonal background of gamma globulins seen with restricted polyclonal gammopathies.^23^, ^24^ The electrophoretic pattern of our case suggests a restricted polyclonal gammopathy.

The extended immunofixation panel used in our case identified the large beta‐gamma peak as IgG4 and identified a polyclonal IgG response comprised of other IgG subclasses. Although IF does not allow direct quantification, correlation with the electrophoretogram can inform a semiquantitative assessment of protein abundance. In our case, it suggests a substantial component of IgG4, which resolved with treatment. A markedly increased IgG4 fraction is thought to be common in humans with IgG4‐RD, but is variable and may be present in only 51% of cases.^17^, ^25^ Canine IgG4 ELISA assays have been developed using either monoclonal or polyclonal reagents, but these are no longer available.^11^, ^26^, ^27^ Although IF can be used to suggest the presence of increased amounts of IgG4, re‐institution of an ELISA assay for IgG4 may facilitate diagnosis of future cases of IgG4‐RD.

Human patients with IgG4‐RD typically are highly responsive to glucocorticoids, if treatment is initiated before the onset of substantial fibrosis.^28^ Treatment is expected to include both resolution of mass‐like lesions and a decrease in serum IgG4 concentration, but, serum IgG4 concentrations can remain high after treatment.^25^ Recrudescence of disease is expected in affected humans but has not been observed in our case, at the time of writing.^17^, ^25^, ^28^ Monitoring serum IgE and IgG4 concentrations has been proposed to help predict disease relapse in affected human patients and could be attempted in affected dogs.^19^

The clinical findings and response to treatment in our case support a diagnosis of IgG4‐RD. The mass‐like lesions, plasmacytosis, and marked hyperglobulinemia with restricted polyclonal gammopathy, which could be interpreted as a monoclonal gammopathy all raised concern for an immunoglobulin‐secreting neoplasm. Previous cases of IgG4‐RD in dogs may have been misclassified as a neoplastic process, as has been documented in human medicine. Clinical awareness of this disease entity in the dog as a differential diagnosis is important when evaluating patients with possible monoclonal gammopathy, plasmacytosis, and eosinophilia.

## CONFLICT OF INTEREST DECLARATION

Authors declare no conflict of interest.

## OFF‐LABEL ANTIMICROBIAL DECLARATION

Authors declare no off‐label use of antimicrobials.

## INSTITUTIONAL ANIMAL CARE AND USE COMMITTEE (IACUC) OR OTHER APPROVAL DECLARATION

Authors declare no IACUC or other approval was needed.

## HUMAN ETHICS APPROVAL DECLARATION

Authors declare human ethics approval was not needed for this study.

## Supporting information


**Appendix S1.** Supporting Information.
