# Supplementary material for: Immunoglobulin G4‐related disease in a dog
Source: J Vet Intern Med. 2019 Oct 26;33(6):2732–8. doi: 10.1111/jvim.15624 (PMC6872619; doi:10.1111/jvim.15624)
Supplement: Supplementary file 1 — Appendix S1. Supporting Information. [file JVIM-33-2732-s001.pdf]

IgG4-Related Disease in a Dog

Authors: Lydia J. Colopy[1], Kai-Biu Shiu[1], Laura A. Snyder[2], Anne C. Avery[3], Emily D. Rout[3], A Russell Moore[3]

1 – VCA Veterinary Emergency Service, Veterinary Specialty Center, Middleton, WI

2 – Marshfield Labs, Veterinary Services, Marshfield, WI

3 – Department of Microbiology, Immunology, and Pathology, College of Veterinary Medicine and Biomedical Science, Colorado State University, Fort Collins, CO, US

Supplemental material

Capillary zone electrophoresis – Marshfield

Capillary zone electrophoresis (CZE) SPE was performed using the CAPILLARYS2 FLEX PIERCING system (Sebia, France), with Sebia Capillarys Protein 6 Kit (Sebia, Cat. #2003, Sebia, France) as described previously.[1] Electrophoretic analysis was performed using Phoresis CORE software (Sebia, France). Controls utilized for testing include Sebia Normal Protein Electrophoresis Control 1 and 2 (Sebia, Cat. #: 4785 and 4787 respectively, Sebia, France).

Urine Agarose Gel Electrophoresis – Marshfield

Urine protein electrophoresis was performed using amido black stained agarose gel electrophoresis (Sebia Hydrasys with Hydragel B1-B2 15/30 with amido black kit, Sebia, France), GELSCAN scanner (Cat. # PN 1206, Sebia, France) and Phoresis CORE software (Sebia, France), as described previously.[1] Patient urine was concentrated X50 or to a maximum of 8000 mg/dL using BJP10 Static Concentrator (Vivaproducts Inc., Cat. #: BJP-5/100, Littleton, MA). Controls utilized for testing include Sebia Normal Protein Electrophoresis Control 1 and 2 (Sebia, Cat. #: 4785 and 4787 respectively, Sebia, France).

Serum Agarose Gel electrophoresis – Colorado State University

Agarose gel electrophoresis (AGE) SPE was performed using amido black stained agarose gel electrophoresis (Sebia Hydrasys with Hydragel Protein (E) with amido black kit, Sebia, France), a flat-bed scanner (Epson Perfection V700 Photo, Epson America, Inc., Long Beach, CA, USA) and Phoresis software (version 8.6.3, Sebia, France), as described previously.[2,3] A pooled canine serum sample from healthy patients previously collected for reference interval evaluation was used as a control for experiments and to aid in assigning fraction demarcations.

Immunofixation – Colorado State University

Immunofixation was performed using the Hydragel IF/Bence Jones kit (Sebia, France), and anti-canine antibodies [anti-canine whole serum (Bethyl A40-108, Bethyl laboratories Inc, Montgomery, TX, USA), anti-canine IgG Fc (Sigma SAB3700101, Sigma-Aldrich, St Louis, MO, USA), anti-canine IgA (Bethyl A40-104A), anti-canine IgM (Bethyl A40-116A), anti-canine light chain (Bethyl A40-124A), polyclonal anti-canine IgG1 (Bio-Rad AHP947, Bio-Rad, Hercules, CO, USA) and polyclonal anti-canine IgG2 (Bio-Rad AHP948)]. Briefly, the manufacturer's instructions were followed except that patient serum samples were diluted with provided diluent at 1:16 (whole serum and IgG lanes), 1:8 (IgA, IgM, IgG1, IgG2, and light chain lanes). Antisera was used neat, except anti-canine IgG FC, which was used at 1:4 dilution. The polyclonal anti-canine IgG1 has been shown to label canine IgG4 with this protocol in our lab.[a]

IgE ELISA

Serum IgE concentrations were assessed using a commercial canine specific IgE ELISA kit (E-40E, Immunology Consultants Laboratory, Inc., Portland, OR, USA). Manufacturer's instructions were followed. Samples were diluted with supplied diluent sufficient to produce results within the linear

range of provided standards. Serum from three clinically normal dogs were also tested concurrent with the index case samples and results were reported as fold increase over the average of the control patient results. All samples were evaluated in duplicate and within the same assay run so as to minimize the effects of intra-run and inter-run variation.

Flow cytometry methods:

Samples shipped to the laboratory were sent overnight with cold packs and kept refrigerated until analysis. Samples were analyzed within 72 hours of being obtained from the dog. Spleen and lymph node aspirates were obtained by the submitting clinic by aspirating tissue material into a solution of saline and 10% canine serum. This suspension was centrifuged and re-suspended in 1 mL of lysis buffer (0.15 M NH_4CL, 1 M KHO_3, 0.1 mM Na_2EDTA, 1 M HCL at a pH of 7.2-7.4) for 5 minutes at room temperature to lyse red blood cells. Samples were then centrifuged, lysed a second time and re-suspended in 200 µL of phosphate buffered saline (PBS)-2% fetal bovine serum (FBS). A 96-well plate was used in which 25 µL of cell suspension was added to individual wells plus 25 µL of a cocktail of antibodies (table 1). Samples were incubated for 20 minutes at room temperature and then washed twice in PBS-2% FBS. Samples then were resuspended in PBS-2% FBS with 10 µg/mL of propidium iodide for dead cell exclusion, and analyzed within 1 hour. Samples were analyzed using a 3 laser Coulter Gallios. All data analysis was carried out using Kaluza software (Beckman Coulter).

Supplemental table 1. Flow cytometry antibody panel.

Tube	Antibody specificity and fluorochrome
1	No antibodies
2	CD3-FITC / CD25-PE / CD5-APC / CD8-Alexa 700 / CD4-Pacific Blue

3	Class II MHC-FITC / CD21-Alexa 647 / CD34-PE
4	Class II MHC-FITC / CD18-PE / CD5-APC / CD14 PE-Alexa 750 / CD4-Pacific Blue
5	CD5-FITC / CD45-PE / CD21-Alexa 647

Clones are as follows: CD45 = YKIX716.13, CD18 = YFC118.3 (human CD18), CD4 = YKIX302.9, CD8 = YCATE 55.9, CD5 = YKIX322.3, CD21 = CA2.1D6, CD3 = CA17.2A12, CD14 = TUK4 (human), class II MHC = YKIX334.2, CD34 = 1H6, CD25 = P2A10.

PCR for antigen receptor rearrangements (PARR) methods:

Genomic DNA was extracted using a QIAamp DNA Mini Kit (QIAGEN, Hilden, Germany). Two amplification reactions were performed, using the primer sequences listed in table 2. Complete immunoglobulin heavy chain (IGH) VDJ rearrangements, incomplete IGH DJ rearrangements, and a positive control reaction were amplified in reaction 1. T cell receptor gamma (TRG) rearrangements were amplified in reaction 2. The PCR protocol includes an initial denaturation step at 95°C for 15:00, followed by 10 cycles of 94°C for 0:30, 64-59°C for 0:30 (decreasing by 0.5^{o}C every cycle) and 72°C for 1:30, and 30 cycles of 94°C for 0:30, 59°C for 0:30 and 72°C for 1:30, with a final extension of 72°C for 7:00. Genescan analysis was performed on an ABI 3130xl system (Applied Biosystems, Foster City, CA) using the DS-33 Dye Set (ThermoFisher Scientific, Waltham, MA). The PCR product was diluted ten-fold and 1 µL was added to 14.5 µL HiDi formamide and 1 µL GS600LIZ size standard. Data were analyzed with GeneMarker software (Soft Genetics, State College, PA).

Supplemental table 2. PARR primers for amplification of complete IGH-VDJ rearrangements, incomplete IGH-DJ rearrangements, rhodopsin (RHO) (positive control), and TRG rearrangements.

Primer name	Binding site	Sequence (5' – 3')
Complete IGH-VDJ		
Brd1	IGHV	GGGGAGACCTGGTGAAGCC
Brd2	IGHV	TGGAGTCTGGGGGAGACCT
Brd3	IGHJ	ACCTGAGGAGACGGTGACC
Brd4	IGHJ	TGAGGACACGAAGAGTGAGG
Incomplete IGH-DJ		
Dh21	IGHD	CTGGGGACTTTTTGACAAGG
Dh33	IGHD	GGCCCCAGTTATTGTCAAAG
Dh43	IGHD	AGTGTCCAGCAAGGAGAAGC
Brd3	IGHJ	ACCTGAGGAGACGGTGACC
Brd4	IGHJ	TGAGGACACGAAGAGTGAGG
Rhodopsin (positive control)		
RhoceF	RHO	ACCACCCAGAAGGCTGAAA
RhoceR	RHO	CTGGGAGGGTCATGAAGATG
TRG		
Jgg1	TRGV	CCCTCCTGTTTCCCGGTA
Dpd	TRGV	ARGCCATGTACTACTGTKCCTG
Jgg12	TRGV	CCCCAGGCACTTCAGTCTAC
Jgg7	TRGJ	TAACCCTGAGMAYTGTGCCA
Dpg	TRGJ	TAACCMTGAGCTTTGTGCCA
Dpf	TRGJ	CCTTGTCCAAATATCTTGATCCA

Jgg11	TRGJ	MCTTCTGTAAATWTCTTGATCCA

Binding sites: IGHV: immunoglobulin heavy chain variable region genes; IGHJ: immunoglobulin heavy chain joining region genes; IGHD: immunoglobulin heavy chain diversity region genes; TRGV: T cell receptor gamma variable region genes; TRGJ: T cell receptor gamma joining region genes

Footnotes

a = Donaghy D, Moore AR. Identification of Canine IgG4 by Immunofixation and Commercially Available Antisera. In review *Vet Immunol Immunopathol*.

References

1. Giordano A, Paltrinieri S. Interpretation of capillary zone electrophoresis compared with cellulose acetate and agarose gel electrophoresis: reference intervals and diagnostic efficiency in dogs and cats. *Vet Clin Pathol*. 2010;39(4):464-473.
2. Errico G, Giordano A, Paltrinieri S. Diagnostic accuracy of electrophoretic analysis of native or defribrinated plasma using serum as a reference sample. *Vet Clin Pathol*. 2012;41(4):529-540.
3. Harris RAD, Rout E, Avery A, Bolte D, Belling-Kelly E, Moore AR. Validation and method comparison of the use of densitometry to quantify monoclonal proteins in canine sera. *Vet Clin Pathol*. In Press.
